# Prices, peers, and perceptions (P3): study protocol for improved biomass cookstove project in northern Ghana

**DOI:** 10.1186/s12889-018-6116-z

**Published:** 2018-10-29

**Authors:** Katherine L. Dickinson, Maxwell Dalaba, Zachary S. Brown, Rex Alirigia, Evan R. Coffey, Elise Mesenbring, Manies Achazanaga, Desmond Agao, Moro Ali, Ernest Kanyomse, Julius Awaregya, Clifford Amoah Adagenera, John Bosco A. Aburiya, Bernard Gubilla, Abraham Rexford Oduro, Michael P. Hannigan

**Affiliations:** 1 Colorado School of Public Health, University of Colorado Anschutz Medical Campus, 13001 E 17th Pl, Aurora, CO 80045 USA; 2 Navrongo Health Research Centre, Behind Navrongo War Memorial Hospital, Navrongo, Ghana; 3 North Carolina State University, Campus Box 8109, 2801 Founders Drive, Raleigh, NC 27695 USA; 4 University of Colorado Boulder, College of Engineering and Applied Science, 1111 Engineering Drive, 422 UCB, Boulder, CO 80309-0422 USA; 5 Organisation for Indigenous Initiatives and Sustainability Ghana, Post Office Box 1, Page, U.E, Navrongo, Ghana

**Keywords:** Cookstoves, Household air pollution, Behavior change, Global health, Study protocol

## Abstract

**Background:**

Despite their potential health and social benefits, adoption and use of improved cookstoves has been low throughout much of the world. Explanations for low adoption rates of these technologies include *prices* that are not affordable for the target populations, limited opportunities for households to learn about cookstoves through *peers*, and *perceptions* that these technologies are not appropriate for local cooking needs. The P3 project employs a novel experimental design to explore each of these factors and their interactive effects on cookstove demand, adoption, use and exposure outcomes.

**Methods:**

The P3 study is being conducted in the Kassena-Nankana Districts of Northern Ghana. Leveraging an earlier improved cookstove study that was conducted in this area, the central design of the P3 biomass stove experiment involves offering stoves at randomly varying prices to peers and non-peers of households that had previously received stoves for free. Using household surveys, electronic stove use monitors, and low-cost, portable monitoring equipment, we measure how prices and peers’ experience affect perceptions of stove quality, the decision to purchase a stove, use of improved and traditional stoves over time, and personal exposure to air pollutants from the stoves.

**Discussion:**

The challenges that public health and development communities have faced in spreading adoption of potentially welfare-enhancing technologies, like improved cookstoves, have highlighted the need for interdisciplinary, multisectoral approaches. The design of the P3 project draws on economic theory, public health practice, engineering, and environmental sciences, to more fully grasp the drivers and barriers to expanding access to and uptake of cleaner stoves. Our partnership between academic institutions, in the US and Ghana, and a local environmental non-governmental organization creates unique opportunities to disseminate and scale up lessons learned.

**Trial registration:**

ClinicalTrials.gov NCT03617952 7/31/18 (Retrospectively Registered).

**Electronic supplementary material:**

The online version of this article (10.1186/s12889-018-6116-z) contains supplementary material, which is available to authorized users.

## Background

Low adoption rates of technologies with the potential to improve public health have been observed in a number of cases across a variety of contexts; examples include bed nets [1], latrines [2], deworming drugs [3], and condoms [4], among many others. Explanations for this phenomenon tend to focus on three key factors: the *prices* of these technologies and the role of subsidies [1, 5, 6], the effect of *peers* and social learning [3, 7, 8], and the ways in which users’ *perceptions* of technologies are influenced by different factors and affect subsequent adoption decisions [3, 6, 9]. The aim of this study is to investigate the interactions among these three factors in determining adoption of improved cookstoves, a technology with potential public health, social, and environmental benefits.

Cooking with biomass over open fires is a widespread practice throughout much of the developing world. Wood, dung, agricultural residues, and charcoal produce large amounts of respirable particles, carbon monoxide, and other toxic pollutants when used to fuel simple cooking stoves [10]. A growing body of evidence links household air pollution (HAP) to acute lower respiratory infections in young children and chronic obstructive pulmonary disease and lung cancer (for coal) in adults [11–13]. Biomass cooking also impacts regional and global climate through black carbon particulates and other emissions [14]. Furthermore, gathering fuels is a time-consuming activity in locations where environmental damage has often already made resources scarce. This time burden, which falls disproportionately on women, could be better spent on domestic care or income-generating activities, aggravating the problem of “time poverty” [15].

While a multitude of technologies exist that could potentially address the suite of problems linked to current biomass cooking practices, efforts to disseminate these technologies and promote changes in cooking behaviors have often fallen short [16, 17]. The Global Alliance for Clean Cookstoves, a public-private partnership currently in its second phase of “investment and innovation,” has set a goal to foster the adoption of clean cookstoves and fuels in 100 million households by 2020 [18]. However, consistent adoption of cleaner stoves has proven elusive in practice at larger, community-level scales. The well-known RESPIRE study provided an improved chimney woodstove to households in highland Guatemala and saw encouraging results, finding a significant reduction in carbon monoxide exposure for groups receiving the clean stove over an 18 month period [19]. On the other hand, randomized trials of a locally-made mud stove in India achieved disappointing initial adoption and maintenance rates and, in the long run, failed to reduce exposure to dangerous air pollutants [16]. These authors specifically contrasted their intervention with the RESPIRE study and argued that they provided households with greater ability to reveal their valuation in usage rates: stoves were locally made and significantly cheaper, were not inspected weekly [20], and were followed for a longer period of time. In response, the RESPIRE study’s lead investigator argued that the Indian “improved” stove was not truly an improvement over existing technologies since it failed to alter combustion and reduce smoke in any meaningful way [21]. Essentially, both sides of this debate contended that *low perceived benefits* of the cookstove technology led to low adoption and use. The cookstove example thus presents itself as a useful context for examining the challenges and dynamics of technology adoption.

### Prior research on technology adoption

Technology adoption continues be a central research topic in public health and social sciences because of its importance in understanding development and health outcomes and because of the kaleidoscope of models explaining different economic, psychological, and sociological factors at play. Two key strands of literature we summarize here examine the roles of prices and peer effects on technology adoption.

#### Prices and technology adoption and use

Setting subsidy and end-user price levels for a new technology requires grappling with a fundamental tension between rapid diffusion and sustainability [22, 23]. On the one hand, subsidizing adoption of socially beneficial technologies may be necessary to promote widespread adoption, at least in the short-run. Indeed, recent evidence has shown that new technologies offered at a positive price tend to exhibit much lower demand than identical products offered for free [24, 25]. In one example that is particularly relevant for this study, Mobarak and coauthors [23] analyze a field experiment with the distribution of cookstoves in Bangladesh. The researchers find demand for these modern stoves to be extremely price elastic, with only 5% of households purchasing the stoves with no discount and a 50% discount yielding 8–12% higher demand (relative to the full cost treatment).

On the other hand, many argue that goods given away for free or at low cost will be *used* at lower rates than goods for which users pay higher prices. Economic theory offers at least two mechanisms for this hypothesis. First, price-based incentives for new technologies (or any scarce good) ensure allocation of goods to those valuing them the most (a basic principle in economics). Second, higher prices may lead potential users to perceive that a product is of higher quality [26], thus encouraging higher use. Empirically, however, there is little evidence to support this hypothesized positive relationship between price and technology use. In one of the few studies to directly test this hypothesis, Cohen and Dupas [1] analyze data from a randomized controlled trial of bednet distribution in Kenya in which health clinics distributed nets freely or partially subsidized at four different end-user price levels (between $0.15 and $0.60 per net). The researchers identify significantly price-elastic demand for bednets: Clinic patients charged the highest price in the experiment exhibited 60% lower demand for bednets relative to the free distribution group. Moreover, despite thorough statistical analysis, Cohen and Dupas do not find evidence that the free distribution group exhibits lower usage rates (conditional on ownership) than the partially subsidized groups. Furthermore, the free distribution group is the only treatment group for which the researchers find a statistically significant health impact (reduced anemia). To our knowledge, these authors did not directly examine the relationship between price and perceived quality of bednets as an intermediate factor affecting product use.

Thus, empirical evidence to date seems to indicate that highly subsidized or free distribution of health-promoting technologies: a) may be required to promote their initial adoption, and b) does not appear to reduce subsequent technology use (although the latter finding has a thinner evidence base and should be tested more broadly). Yet free distribution strains public resources and may not be sustainable over time or scalable to population-level technology diffusion. Additional work is thus required to examine the dynamics of diffusion over time and space. One particular question involves the possibility that subsidizing adoption to an initial group of users can lead that group’s peers to learn about and subsequently adopt a technology and, assuming the technology is useful, positively affect individuals’ willingness to pay (WTP) for the technology.

#### Peer effects and technology adoption

Peer effects present the possibility of a positively reinforcing feedback for sustaining adoption and takeoff of new technologies. The power of social contagion in technology adoption has been measured in a number of contexts [e.g., 27]. Miller and Mobarak [9] estimate peer effects on efficient cookstove adoption in Bangladesh, by conducting randomized, sequential cookstove rollout first with opinion leaders, then with a first round of randomly selected members of the general population (in the same neighborhoods as the opinion leaders), and then with social contacts of the first round households. Their results suggest statistically significant and positive peer effects from opinion leaders’ adoption behaviors (at least in some cases), but social ties to first round participants are found to *reduce* the likelihood of adoption among second round households. The authors’ interpretation of this finding is that second round participants held initially high expectations about the modern stoves, and revised these expectations downward via information from social contacts. This negative peer effect finding and its interpretation are similar to Kremer and Miguel’s [3] analysis of deworming drugs in Kenya. Yet to our knowledge, neither study explicitly measured expectations or beliefs about product quality. Both of these cases highlight the fact that while the increasing availability of experimental data and appropriate econometric methods for analyzing these data have gone a long way toward solving Manski’s [28] “reflection problem” and enabling identification of peer effects, this research has also raised a number of new questions about the causal mechanisms underlying observed effects.

In light of the previous research outlined above, we aim to contribute to a more scientific understanding of the interactions between economic incentives (“prices”), social learning (“peers”), and subjective beliefs (“perceptions”) in technology adoption dynamics. Specifically, we posit that prices and peer effects both operate – at least in part – through separate and interactive effects on perceptions of a technology’s quality and benefits.

### Conceptual model

**Fig. 1:**
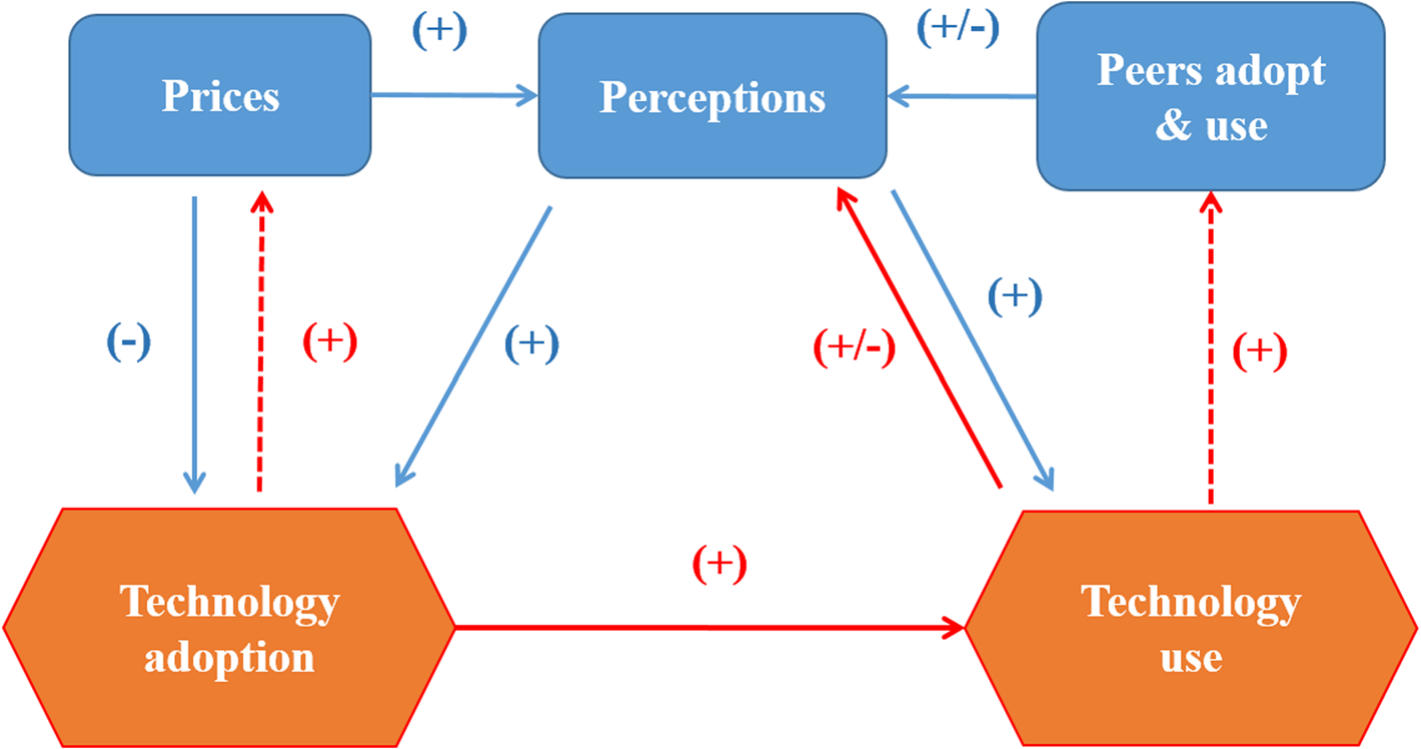
Influence diagram showing technology adoption dynamics. The solid arrows in the diagram are influences that this study will examine in detail. The dashed arrows are potential confounding feedbacks that our identification strategy will address. The signs in parentheses indicate whether effects are expected to be positive or negative, based on previous literature

Figure 1 presents our conceptual model using an influence diagram of how we expect prices, peers, and perceptions to interact, based on previous research. Prices can be expected to have both direct and indirect influences on key outcomes (technology adoption and use): The direct effect (the economic “law of demand”) is expected to be negative, while it is possible that there is a positive indirect effect on both adoption and use via higher perceptions of technology benefits for higher-priced products. Peer effects can be expected to affect individual adoption and use through effects on individuals’ perceived value of the new technology. This effect can be negative or positive.

Importantly, the conceptual model in Fig. 1 also highlights the potential feedbacks (the dashed arrows) that can confound causal identification, and which our experimental design seeks to address. First, a number of factors determine prices for a new technology in an observational setting, including supply and retail costs. We will address this confounding feedback using prices which are randomly assigned across groups of households. Second, peer effects are well-recognized for their potential to generate positive feedback loops. We will control for this confounder by sampling households neighboring participants in the previous study’s cookstove intervention, in conjunction with the recruitment of new groups of households unexposed to the technology. This identification strategy for peer effects appears unique compared to previous research [3, 9, 29].

Finally, an important question for sustainability science and public health is how subjective expectations change following technology adoption and subsequent use, and how these revised expectations determine long-term use. For example, we might hypothesize (e.g. based on the Prospect Theory literature [30]) that discovering a new technology to yield smaller than expected benefits may be have a greater downside effect on usage than the upside effect of finding the technology to have greater than expected benefits.

Additional key questions emerging from this model are how the individual factors affecting key outcomes of interest are mediated by the other factors. A standout issue along these lines is the possibility that peer effects may dampen the role of prices in subjective perceptions of technology quality. This is one hypothesis suggested by Ashraf et al. [6], who conducted an information-based interventions in the case of improved water filter subsidization in Zambia and found that information provision increased the price elasticity of demand, making price subsidies more effective. The authors remain agnostic on the causal mechanisms behind this finding, but suggest that uninformed consumers may use price as an indicator of product quality.

## Methods and study design

### Study area

**Fig. 2:**
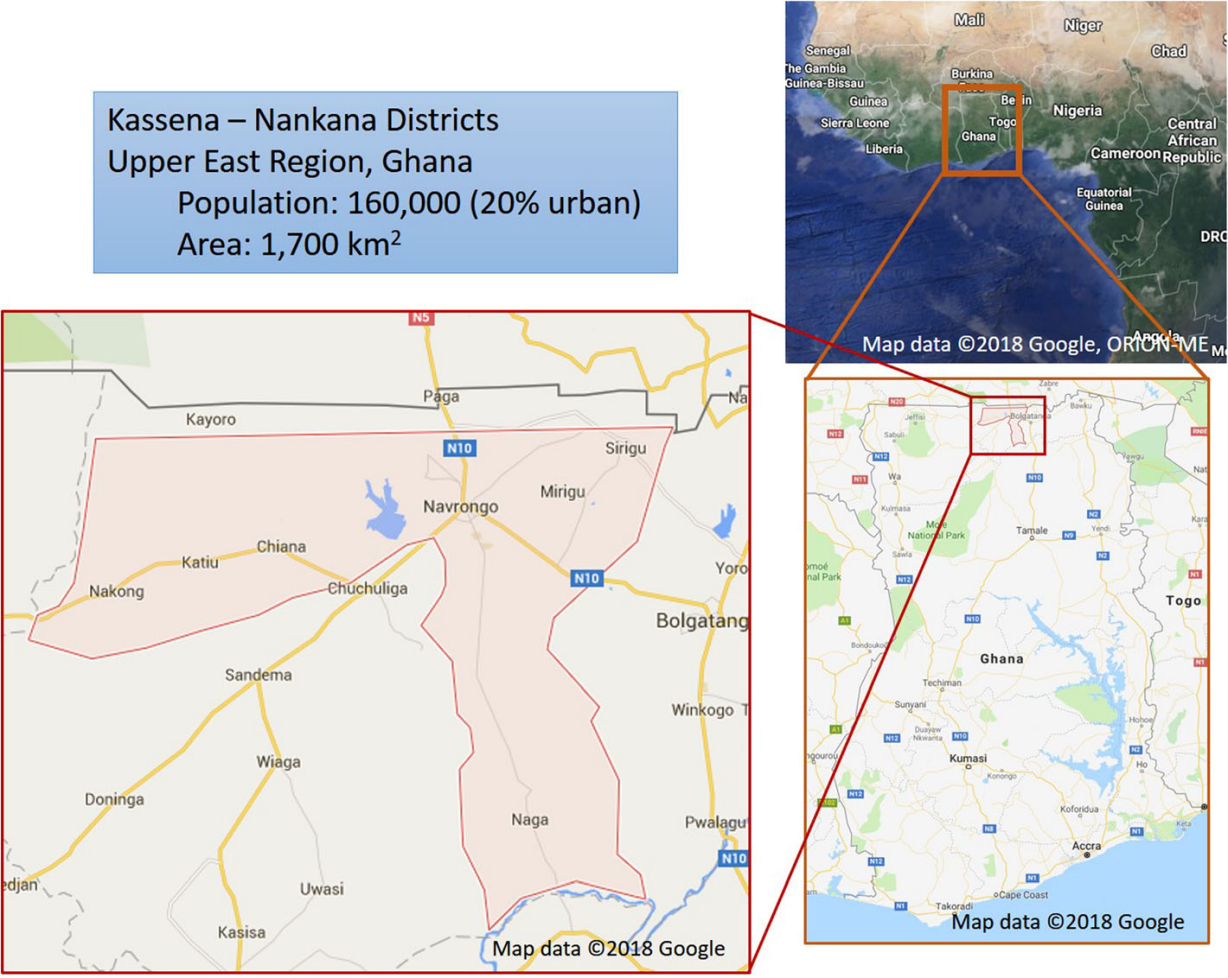
Map of the study area. Source: Authors’ creation with map and imagery data from Google, ORION-ME, Data SIO, NOAA, U.S. Navy, NGA, GEBCO, Landsat/Copernicus, U.S. Geological Survey, IBCAO

The P3 study takes place in the Kassena-Nankana Districts (KND) in Northern Ghana (Fig. 2). The climate in this region is hot and arid, with one rainy season lasting from approximately May to October, and the vegetation is dominated by woody shrubs and grassland. Much of the land is used in subsistence agriculture, with millet as the dominant crop. Since 1993, the Navrongo Health Research Centre (NHRC) has conducted a district-wide Health and Demographic Surveillance Survey (HDSS) [31]. According to HDSS data, the total population of the KND is about 156,000 (roughly 30,000 households), with about 80% living in areas classified as rural while 20% are in more urban areas, primarily in and around the central town of Navrongo. Eighty eight percent of rural households report using biomass (wood or agricultural waste) as their main cooking fuel, while another 9% rely primarily on charcoal, and only about 3% of households cook primarily with gas or electricity. The traditional cooking method in these rural areas is a three-stone open fire, with many households also using charcoal stoves. Cooking is done both indoors and outdoors. Ghana has one of the highest deforestation rates in Africa with the country’s forest an estimated quarter of its original size [32].

### Prior research: The REACCTING study

The P3 project builds on an earlier cookstove study that our research team conducted in this region, the Research on Emissions, Air Quality, Climate, and Cooking Technologies in Northern Ghana (REACCTING) study [33]. The primary objective of the REACCTING study was to assess the effectiveness, feasibility, and sustainability of scaling up use of improved cookstoves in Northern Ghana through a coupled natural-human systems approach that explores the linkages among human behaviors (i.e., cooking practices), detrimental air quality at multiple spatial and temporal scales, and health outcomes (respiratory illness).

For the purposes of the P3 project, the key feature of the REACCTING study was a randomized household-level intervention which distributed two types of improved biomass stoves for free to 200 participating households in the rural areas of the KND. Based on extensive feedback from households in the KND that tested several stove models during a pilot phase (2012–2013), two different stove technologies were selected for the REACCTING intervention study: the Gyapa Woodstove and the Philips Smokeless Woodstove (HD4012). The Gyapa Woodstove was specifically designed for use by populations in the Northern Regions of Ghana by Relief International/Gyapa Enterprises. A similar model was used in a past intervention study in Accra, and saw significant decreases in kitchen CO and PM2.5 levels [34]. This model includes a combustion chamber, often called a rocket-stove design, with a ceramic liner on the inside and an outer liner of insulation and sawdust to increase heat retention. Meanwhile, the Philips stove was a gasifier stove produced in Lesotho. This stove was visually perceived as “high-tech,” required power (supplied, in our context, through a small solar panel) to perform properly, and had been observed to be a low emitting technology, Tier 3 stove, during lab testing [35].

The target population for the REACCTING intervention study was rural households in the KND that used biofuels (wood, animal waste, and crop residue) as their main cooking fuel source, and that contained women and young children (demographic groups typically in closest proximity to cooking activities). Data from the HDSS enabled a cluster random selection of households from the district population that met the REACCTING eligibility criteria. The social structure in this region is such that groups of related households live in connected compounds. For the purposes of the HDSS, compounds are grouped into geographic clusters. These clusters are grouped into five geographic regions: four of these are primarily rural (North, East, South, and West), while the Central region contains Navrongo town and surrounding areas. For the REACCTING sample, we first eliminated households from the Central region, and then randomly selected 25 clusters using population weighting to determine the number of clusters selected per region. Within each cluster, eight households were randomly selected from the population of households that met the study eligibility criteria, resulting in a total sample of 200 households.

The stove intervention of the REACCTING study included four different intervention arms: Group A received two Gyapa stoves, Group B received two Philips stoves, Group C received one of each type of stove, and Group D served as the control for the duration of the study and received two stoves of their choice at the study’s conclusion. Stove stacking (i.e., households using new cookstoves alongside traditional cooking methods) had been observed in prior studies and we had earlier observed multiple stove use by the households in the study area. Multiple stoves were thus provided to each intervention household to increase the probability that households would begin to substitute away from traditional stoves rather than simply adding a new stove to their cooking technology mix. Randomization into intervention groups was done at the cluster level: i.e., within each of the 25 clusters, there are 2 households in each of the 4 REACCTING intervention groups. Stove distribution for the three intervention arms (A-C) occurred in December of 2013 and January of 2014. The control group (D) received their stoves in mid-2016.

### P3 intervention design

To investigate how prices, peers, and perceptions affect adoption of improved cookstoves, our study leverages the fact that the REACCTING study’s free distribution of stoves to randomly selected households provides those households’ peers with information about these new technologies. Building on this prior work, the P3 study offers new stoves at *different price levels* to groups of households *with and without social ties* to the households that received stoves as part of this prior study. Through these experiments, **we will be able to identify the interacting feedbacks between prices and peer effects on perceptions** of stoves, as well as adoption, use, and personal exposure outcomes across different groups. Our study design is targeted towards these **specific research questions**:

1. How are prior perceptions of the benefits of a new technology affected by the technology’s price? For example, does higher price signal higher quality to target users?
2. How do prior perceptions of a new technology vary based on connections to peers that have experience with that technology? Specifically, how do peers’ adoption and use histories help potential users of a technology learn about product quality?
3. How does peers’ experience influence the relationship between price, on the one hand, and perceptions, technology adoption and use outcomes, on the other? Do peer effects increase or decrease the price elasticity of demand for the new technology?
4. How do perceptions of a technology change over time among households that adopt that technology initially? How do these perceptions relate to objective measures of stove performance (e.g., personal exposure to pollutants), and what is the relationship between perceptions and technology *use* over time?
5. How much cleaner are the improved stoves, operated by end users, than traditional stoves? What emission and pollutant exposure differences exist among the improved and traditional stoves and how does user behavior impact these outcomes?

We address these questions, which are of central interest to the public health community, using interdisciplinary methods, data collection and analysis.

#### Stove selection

The design of our intervention requires that we offer stoves that are similar to those offered for the REACCTING study, since we are measuring whether learning about these technologies through peers influences adoption decisions. However, our experience in the REACCTING study revealed some key challenges with the two specific stove models used in that study (the Gyapa rocket stove and Philips forced draft stove). We thus elected to use slightly different stove models for the P3 project. A review of available technologies and consultation with manufacturers led us to select the ACE1 forced draft stove as a replacement for the Philips. Similar consultations and lab testing at CU Boulder allowed us to narrow our rocket stove options down to two: the Greenway Jumbo and the EcoZoom Dura. A focus group discussion was conducted in September 2016 with participants similar to our target customers to compare and assess preferences for these two models. During the focus group discussion, the team demonstrated the use of these stoves to participants. Participants were then divided into groups and given the necessary ingredients/materials to use the stoves to cook a common dish (jollof rice). Participants gave positive feedback on both stoves, but expressed a slight preference for the Greenway Jumbo, which we subsequently selected for our intervention.

#### Sample selection

**Fig. 3:**
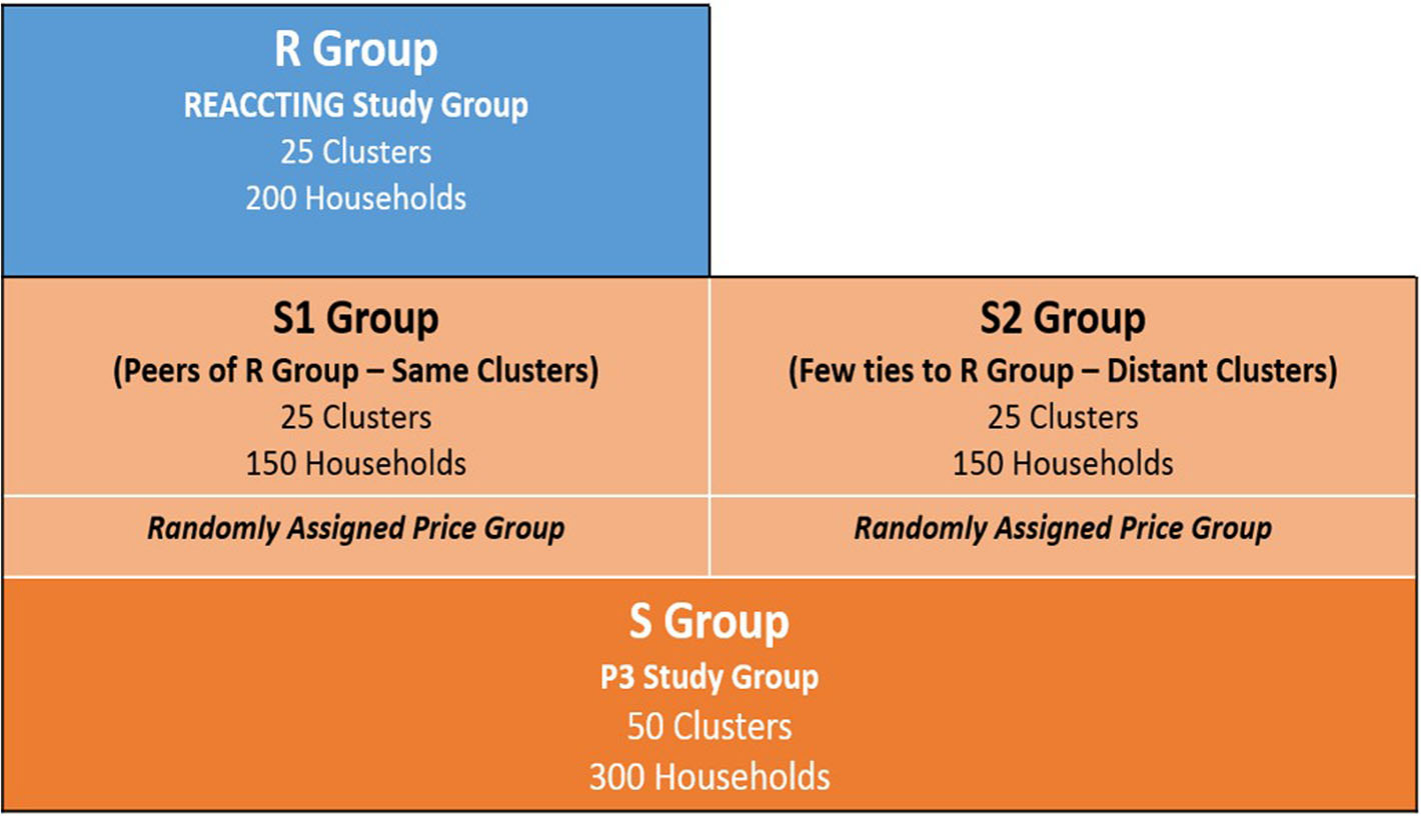
Study design

The study design is summarized in Fig. 3. For the purposes of this design, we refer to the REACCTING study sample as the R Group. Newly enrolled households that are the primary focus of the P3 study, are referred to as the S Group. Our two-phase sample selection procedure involves first selecting clusters, and then selecting households within each cluster. In the first phase, the **S1** subgroup was selected to include the **same clusters** as the R Group households (25 clusters), while the **S2** subgroup consists of 25 clusters randomly selected from the rural areas of the KND outside of a certain buffer distance from the R Group clusters. Given that there are more than 300 clusters in the district and only 25 were included in the R Group, social ties between S2 and R Group households are expected to be minimal (and are measured as part of our data collection).

Next, the required number of **households** was selected from each cluster. We used the same inclusion and exclusion criteria used to select households in the REACCTING study (i.e., rural, using biofuel, having one woman 18–55 and one child under 5). S1 group households were selected as *nearest eligible neighbors* of each of the 6 REACCTING intervention households in each cluster. In the non-peer clusters, 6 seed households, each meeting the above eligibility criteria, were randomly selected, and then non-peer group households were selected as the nearest neighbors of those seed households.

By using a uniform set of selection criteria and sampling methods between the peer and non-peer groups, and given that both the R/S1 group and the S2 group clusters were randomly selected, the study design ensures that in expectation the only differences between S1 and S2 group households is the former’s higher level of contact with peers that have cookstove experience, enabling us to test the impacts of peers on our outcomes of interest (perceptions and technology adoption and use).

#### Setting stove prices

To examine the effects of price (and the interactive effects of prices and peers) on perceptions and technology adoption, both S1 and S2 Groups were randomly subdivided into multiple price treatment groups. The price randomization is done at the cluster level – i.e., all households in a cluster are offered stoves at the same price.

In order to generate variation in stove purchasing behavior that we can use to assess impacts of prices and peers, we require information on an approximate range for households’ willingness to pay (WTP) for the different stove models in the study population. Estimates of WTP come from two primary sources. First, during the REACCTING study, we measured participants WTP for improved stoves at multiple time points. During the study’s baseline survey, a choice experiment was conducted to assess stated WTP for hypothetical stoves with different attributes (e.g., less smoke, faster cooking time relative to traditional stoves). These stated WTP values were quite high; for example, average WTP for stoves that produced less smoke was on the order of 200 GHC (~USD$50) [36].

**Fig. 4:**
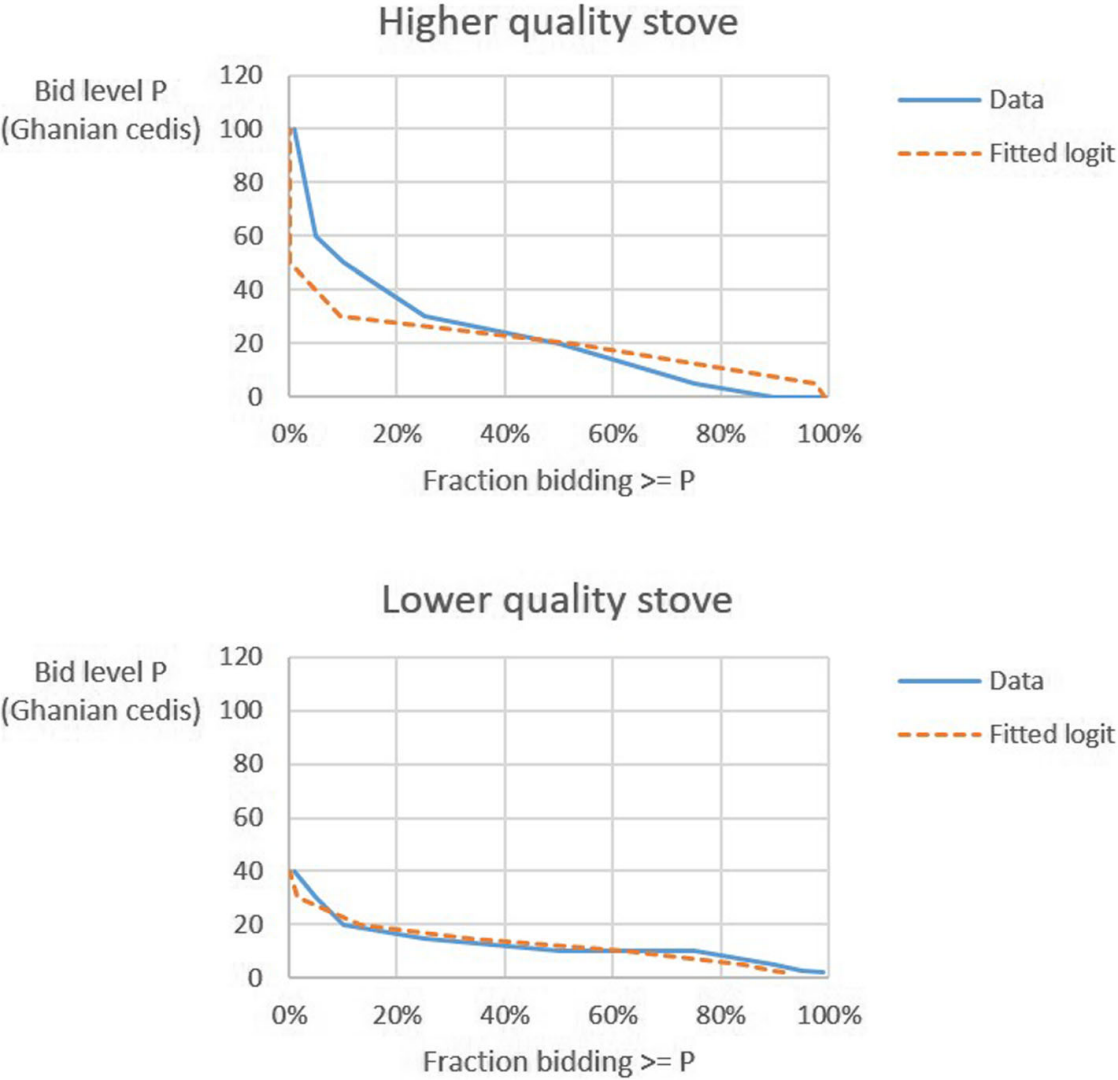
Distribution of bids for the higher quality (upper panel) and lower quality (lower panel) stoves in the stove auctions

Due to concerns that these stated WTP values may have been larger than households’ true willingness and ability to pay for improved stoves in this area, we decided to collect revealed preference information on WTP during the P3 design phase. Specifically, in November of 2015 we conducted a series of five focus group discussions (FGDs) in which we conducted a 2nd price, sealed-bid auction of different stove models. Under classical economic assumptions, participants should bid their true ex ante WTP for the good [37]. The bid data from these auctions therefore provide some guidance on the range of households’ WTP for different stove models. We auctioned one “mid/low-quality” stove – the Gyapa stove used in the REACCTING study – and two “high-quality” stove models – the ACE1 and the Philips. The mean bid for the higher quality stoves was 67% higher than for the Gyapa (Table 1). A quarter of participants in the higher-quality stove auctions bid at least 30 cedis, whereas only 5% of participants in the lower-quality stove auctions bid at least this amount (Fig. 4).

**Table 1:** Bid amounts for three types of stoves sold in auctions

Stove	Number of Bids	Bids (Ghanian cedis)	
		Mean	Std. Dev.
Gyapa	31	13.10	8.19
Philips	23	19.35	16.88
ACE	27	24.04	25.25

#### Randomizing prices across clusters

The experimental design for these interventions involves selecting price levels for the two stoves and distributing these prices across the peer and non-peer clusters. These price levels are set with the aim of maximizing the statistical precision of estimated economic demand for the stoves. The design procedure adopts methods from the economic discrete choice experiment literature, to select price levels which maximize the D-efficiency criterion [38] and uses prior, preliminary information on households’ WTP for the stoves elicited in auctions during the FGDs.

We use a D-efficiency method, which follows the standard principle of seeking a set of experimental treatments which minimize the asymptotic covariance of the treatment effect estimates given a fixed sample size. We follow standard practice in the discrete choice econometric literature and base our D-efficiency design on a conditional logit model [39, 40], in which the probability of an experimental subject selecting stove *j* from a choice set *t* is:

$$ {p}_{j\mid t}\left(\beta \right)=\frac{\exp \beta {x}_j}{\sum_{k\in J}\exp \beta {x}_k} $$

where *x*_*j*_ is a column vector of the stove’s *K* attributes (in our application, price and the stove model) and *β* are regression coefficients to be estimated. D-efficiency seeks to identify a series of choice sets *t* = 1, …, *T* that minimize the expected asymptotic variance of maximum-likelihood estimate (MLE), *β*_*MLE*_. The asymptotic variance of the MLE is inversely proportional to the Fischer information matrix, which in the conditional logit model with *T* choice sets compromised of *A* alternatives each is:

$$ \mathcal{I}\left(\beta |\mathcal{X}\right)=\sum \limits_{t=1}^T{X}_t^{\prime}\left[\operatorname{diag}\left({\boldsymbol{p}}_t\left(\beta, {X}_t\right)\right)-{\boldsymbol{p}}_t{\left(\beta, {X}_t\right)}^{\prime }{\boldsymbol{p}}_t\left(\beta, {X}_t\right)\ \right]{X}_t $$

where *X*_*t*_ is the *K* × *A* matrix of attributes of each alternative in choice set *t*, $\mathcal{X}$ is the collection of these matrices over all *T* choice sets, and ***p***_*t*_(*β*, *X*_*t*_) is the 1 × *A* vector of conditional logit predicted probabilities given regression estimates *β* and attributes *X*_*t*_. (In matrix notation, the diag(*x*) function of a vector *x* forms a square matrix with the elements of *x* along the diagonal and zeros everywhere else, and *X*^′^ denotes the transpose of *X*.) The D-efficiency objective is to find a collection $\mathcal{X}$ of alternatives and attributes which maximize the determinant of $\mathcal{I}\left({\beta}_{MLE}|\mathcal{X}\right)$. In practice, *β*_*MLE*_ is not known a priori, and so an initial guess *β*_0_ is used in experimental design.

**Fig. 5:**
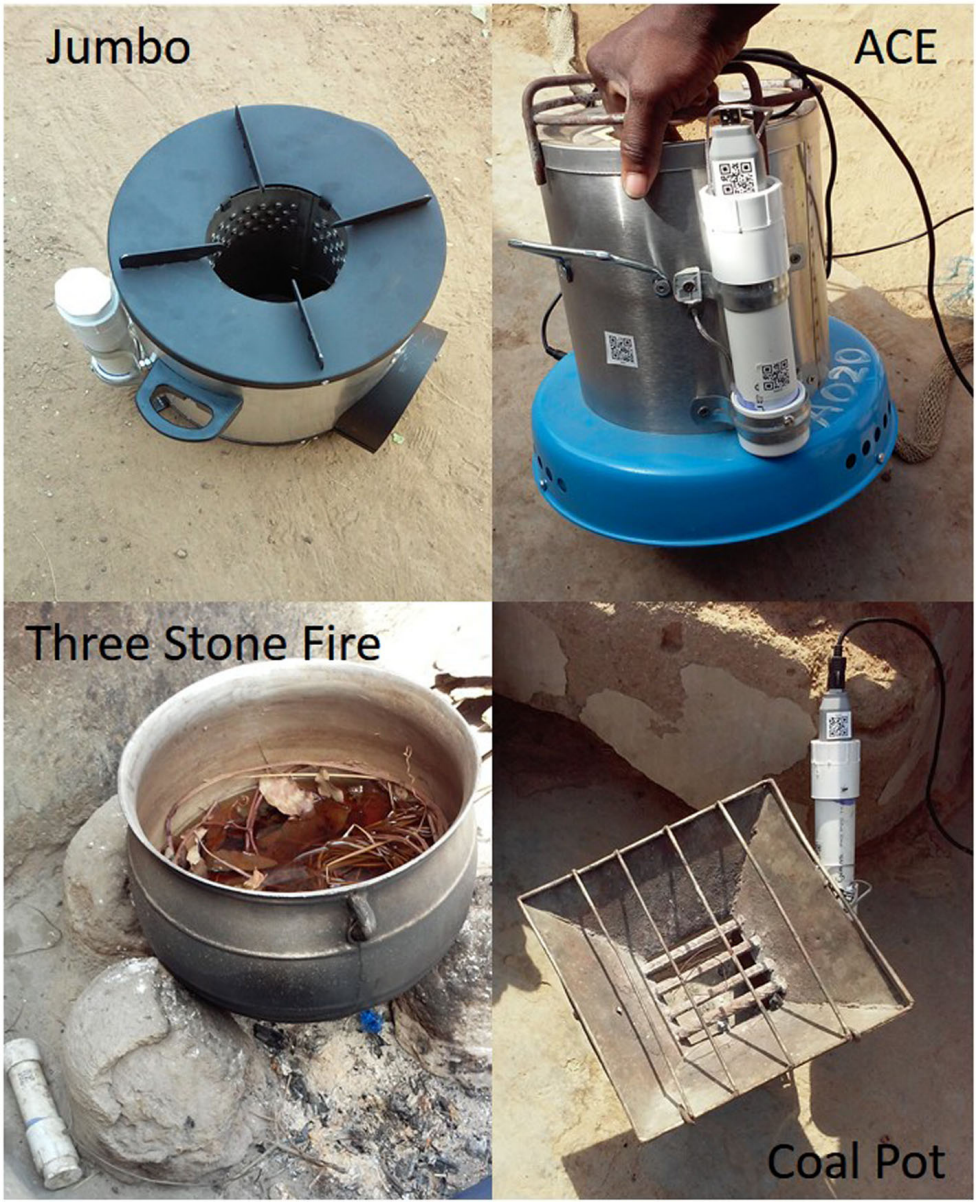
Placement of stove use monitors (SUMs) on improved stoves (Jumbo and ACE) and traditional stoves (Three Stone Fire and Coal Pot). Images source: study authors

Based on study resources, we decided that each household would be offered the option of purchasing up to two stoves consisting of any combination of the higher and/or lower quality stove models at prices randomly assigned to that household’s cluster. Therefore, each choice set consisted of 6 alternatives (1–2 stoves of only one model, 1 of each model, or no stoves), and the only components of $\mathcal{X}$ that were experimentally controlled were these price levels. For logistical ease, we prespecified the possible price levels to be 0, 30, 60, 120 or 240 Ghanian cedis, which encompassed a range of prices from free-distribution to near 100% the cost of the stoves. Following standard practice, dominated alternatives were also eliminated from the design: in our case price configurations in which the lower-quality stove was sold at a higher price than the higher quality stove. The initial guess *β*_0_ of the conditional logit regression coefficients was obtained first from logit curves fitted to the FGD auctions (Fig. 5 above). The experiment was also stratified by study subregion (North, South, East or West), and replicated in S1 and S2 clusters. After launching the sales experiment in the North, we observed higher than expected stove demand, based on the FGD auctions; we therefore redesigned the price treatments again with the D-efficiency method for the remaining three regions based on this higher observed demand.

### Assessment of intervention impacts

In order to measure the impacts of the intervention, we collect data on multiple covariates and outcomes using surveys and other monitoring instruments. The variables that will be measured are summarized in Table 2.

**Table 2:** Summary of measurements to be included in study

Variable	Description	Data Source	Groups Measured	Timing of Measurements
Social Networks	Linkages among and between S Group and R Group households	Surveys	All households (R and S Groups)	Baseline, Endline
Stove Perceptions	Likert-scale and subjective expectation questions measuring perceptions of stove quality / performance for both stove types along multiple dimensions: smoke, fuel use, cooking time, durability, ease of use, suitability for cooking common dishes.	Surveys	All households (R and S Groups)	Baseline, Endline
	Stated willingness to pay / accept for stove types	Surveys	Already measured for R Groups pre-intervention; Measurements added for S Groups	Baseline, Endline
Stove Choice	Number (0, 1, or 2) and type(s) of stoves *selected* and *purchased*	Stove orders and delivery / payment	S Groups R Control Group	Baseline and Stove Delivery
Stove Use	Reported use of all stoves (traditional and improved) in all households on day and week prior to surveys	Surveys	All households (R and S Groups)	Baseline, Endline
	Electronic monitoring of stove temperature	Stove Use Monitors (SUMs)	Subset of stoves & households across all groups	Continuous
Stove Impacts	Kitchen concentrations of and personal exposure to carbon monoxide (CO) and particular matter (PM_2.5_) among study participants	CO and PM monitors	Same households as SUMs (above)	48-h measurements every 3 months
	Self-reported health symptoms	Surveys	All households	Baseline, Endline
	Cost of illness: direct and indirect costs of treating any reported illnesses	Surveys	All households	Baseline, Endline
Household characteristics	Household location, size and demographics, baseline cooking fuel, socioeconomics	HDSS	All households in district	Every 1–2 years
Additional socioeconomic variables	Education, occupation of respondent and household heads Expenditure inventories Agricultural practices	Surveys	All households in study	Baseline, Endline
School attendance	Number of absences for school children enrolled in study	School records	All households in study	Yearly

#### Baseline household survey

For all 300 household participants, we conducted a comprehensive baseline survey between Dec 2016 and Feb 2017. This survey measured household composition and demographics, attitudes and priorities, cooking behaviors (including type(s) of stoves used, fuel use, foods cooked, who cooks within household), knowledge and perceptions of issues related to cooking practices, demand for new stoves, and self-reported health measures. In each household, the primary cook (typically female, aged 18–55 years old) served as the main survey respondent. In households where another male household member makes financial decisions, we also conducted a secondary survey with this individual. All baseline and follow up surveys are conducted using electronic tablets and the Open Data Kit (ODK) software.

#### Perceptions survey

After stove orders were made, but before the recipients received their new stoves, we conducted a short follow up ODK survey with all 300 households to measure their perceptions of the different types of stoves (Jun-Aug 2017). Since our central research questions involve the roles of both prices and peers in shaping stove perceptions, these surveys provide important data on how participants perceive the different stoves and what benefits they expect to derive from them a priori.

#### Endline survey

An endline survey will be conducted with all households in Aug-Oct 2018. This survey will collect information on the same topics measured in the baseline and perceptions surveys. A focus will be on measuring use of both traditional and improved stoves, satisfaction with stoves’ performance, and perceptions of stoves’ impact on household air quality (to be compared with objectively measured air quality and exposure data).

#### Stove orders, payments, and refusals

The intervention is being implemented by a local environmental NGO, the Organization for Indigenous Initiatives and Sustainability (ORGIIS). Between March and May of 2017, ORGIIS and NHRC staff held a series of cluster-level meetings (6 households per cluster) during which they demonstrated the two types of stoves and explained their benefits, and then provided participants the choice to purchase 0, 1, or 2 stoves (total) of either type at the cluster-randomized price levels. Stoves were then ordered from manufacturers and imported; stoves arrived in Navrongo in August, and were distributed to participants in October of 2017. ORGIIS staff will collect payments for stoves over a six month period, with first payment due at the time of delivery. (This payment arrangement was explained during the stove offer meetings, so households were able to make their purchasing choices with this information.) ORGIIS staff are also trained in stove maintenance and repairs, and are available to troubleshoot any problems that households face in using their stoves. In addition, follow-up visits are being conducted periodically to encourage households to use their stoves and assess any challenges users are facing.

Detailed data on stove orders and payments are being collected and shared with the research team. This includes any instances in which households who initially ordered stoves decide not to follow through with their purchases at the time of delivery, or fail to make all necessary payments within a six month period (in which case stoves will be recovered by ORGIIS, and any payments made by the household will be returned).

#### Stove use monitors

Out of the 300 household participants, a subset of about 50 households will have their stove use monitored continuously throughout the follow up period using electronic Stove Use Monitors (SUMs). These households have been selected to represent variation in bid outcomes (number and types of stoves chosen) as well as price levels and peer groups.

The SUMs units we have developed for this project consist of thermocouple data loggers (Thermocouple Temperature Data Logger SSN-61, Wellzion), Type K thermocouples (1 M K Screw Thermocouple and 2 M Customized K Thermocouple, Wellzion), and PVC enclosures that protect the units from water and heat exposure. The total unit cost is ~USD$25. The SUMs have a measurement range of approximately − 270 °C to 1200 °C, a substantially larger range than devices such as the iButton or Labjack Digit SUMs that have been frequently used in previous cookstove studies [41] and operate from about − 40 °C to 85 °C. The higher upper limit operating temperature allows us to place these SUMs as close to the hottest portion of the cookstove as desired without concern of overheating, resulting in clearer designations between heating due to the sun or ambient temperature rises and actual cooking events.

In each of the 50 selected SUMs households, all stoves within the household (improved and traditional) will be equipped with SUMs. Different types of stoves require slightly different SUMs placement to optimize data quality and cooking event detection (see Fig. 5). For the improved stoves, SUMs units are secured to the side using metal tube brackets screwed into the stove, and the thermocouple probe is screwed directly into the tapped metal side of the stove. Placement for traditional stoves (charcoal stoves and three stone fires) is somewhat more complicated given variation in stove designs throughout the study area. Examples of placements for each of these stoves are shown in Fig. 5. SUMs measurements of three stone fires have proven particularly challenging in previous work [42]. Our protocol for these stoves involves placing the probe within six inches of the center of the fire, securing the probe and wire with multiple 6″ long ground staples, and extending the PVC enclosure away from the stove and covering it with stones or other objects to stabilize and secure the unit. Pre-testing was completed to ensure that the probe type and placement on each stove type yielded a large enough temperature increase when the stove was ‘on’ to recognize a cooking event.

SUMs units are set to log a temperature reading every five minutes. Using this logging interval, SUMs have enough memory to log for 111 days, so team members visit these households to download data at least once every three months. Units are equipped with a watertight screw-top adapter that allows the field team to remove the SUM and download data in the field. Each SUM and stove is labeled with a QR code sticker, which allows us to keep track of which SUM corresponds to which stove as well as which stoves are in each household. At each data download visit, an electronic survey (ODK) is completed to record reported stove use, any issues with the stove, stove location, fuel use, and any problems with the SUMs.

#### Personal exposure and household air quality

Expanding on previous personal exposure and household air quality assessments completed in the KND [43, 44], this study aims to further quantify the effects of new stove technologies on personal exposure and household air quality and explore relationships between the two. Personal exposure and household air quality will be measured in a subset (*n* = 40) of households receiving SUMs. Biweekly, eight households are visited for exposure sampling: four from Monday to Wednesday and another four from Wednesday to Friday. This sampling allows 2–3 repeat visits to each household over the follow up period to explore within-household variation.

Personal exposure to carbon monoxide (CO) and particulate matter less than 2.5 μm in diameter (PM_2.5_) are measured using near real-time CO loggers (EL-USB-CO ~USD$125, Lascar Electronics) and light scattering PM_2.5_ sensors (HAPEx Nano ~USD$120, Climate Solutions Consulting). Each device is set to store data with one-minute time resolution although longer sampling intervals are optional. The HAPEx averages three 20-s samples for each minute reading. Monitors are affixed to lanyards or waist packs and worn by the primary cook and, if present, children under the age of 5 for a duration of 48 h to account for day-to-day variability. Young children are given custom t-shirts with pockets sewn on the lapel to hold the instruments.

The Lascars and HAPEx loggers represent some of the strongest exposure monitoring tool candidates balancing price, size and data quality. Lascar loggers are one of the most prevalent CO monitoring tools in personal exposure assessment due to the ease of use and general reliability. They have been found to be reasonably accurate and precise yet require calibration before use and continued characterization over time due to sensitivity changes and response time lags, due in part to sensor fouling [44]. The Lascars house electrochemical sensors which are calibrated in the lab using a normalization technique and at the field site every 2 months using zero air and span gases. The HAPEx Nano (Climate Solutions Cons.) instrument is a proxy for PM_2.5_ and incorporates the Sharp GP2Y sensor. The Nano has been found to be one the highest performing low-cost portable sensors for exposure assessment during lab tests and, more importantly, in field validation [45]. The Nanos are zeroed once a week in a clean chamber and rotate through a 48-h collocation with a set of pump and filters at a focus household (described below). These cumulative PM_2.5_ filter measurements act as a reference with which to normalize the Nano readings. The HAPEx Nano also employs an accelerometer to measure compliance.

Personal exposure has been found to be highly dependent on a participant’s time-activity [46–50]. In order to gain a better understanding of the exposure incurred from each stove type, proximity monitors are employed at each deployment household in the primary and secondary kitchen areas. Using a network of Bluetooth Low Energy (BLE) emitters (beacons worn by participants, Roximity) and receivers (Android cell phones positioned next to the cookstoves), distance to cooking areas can be estimated using registered signal strengths indications (RSSI) akin to the method conducted in REACCTING and similar to other time-activity monitoring approaches [51]. Coupled with SUM information and proximity data, exposure to CO and PM_2.5_ can be apportioned to various activities and stove types. Moreover, monitoring a subset of participant’s location using a GPS-enabled watch (Suunto Ambit3 Peak) offers participant time-activity information for validation of measured proximity categories (e.g., at home, away from home) and the ability to indicate exposure incurred outside the household, a potentially large fraction of total exposure [43, 44, 52]. Proximity information will also be integrated with SUM data to explore how cooking behavior (e.g., time tending fire) changes based on stove.

**Fig. 6:**
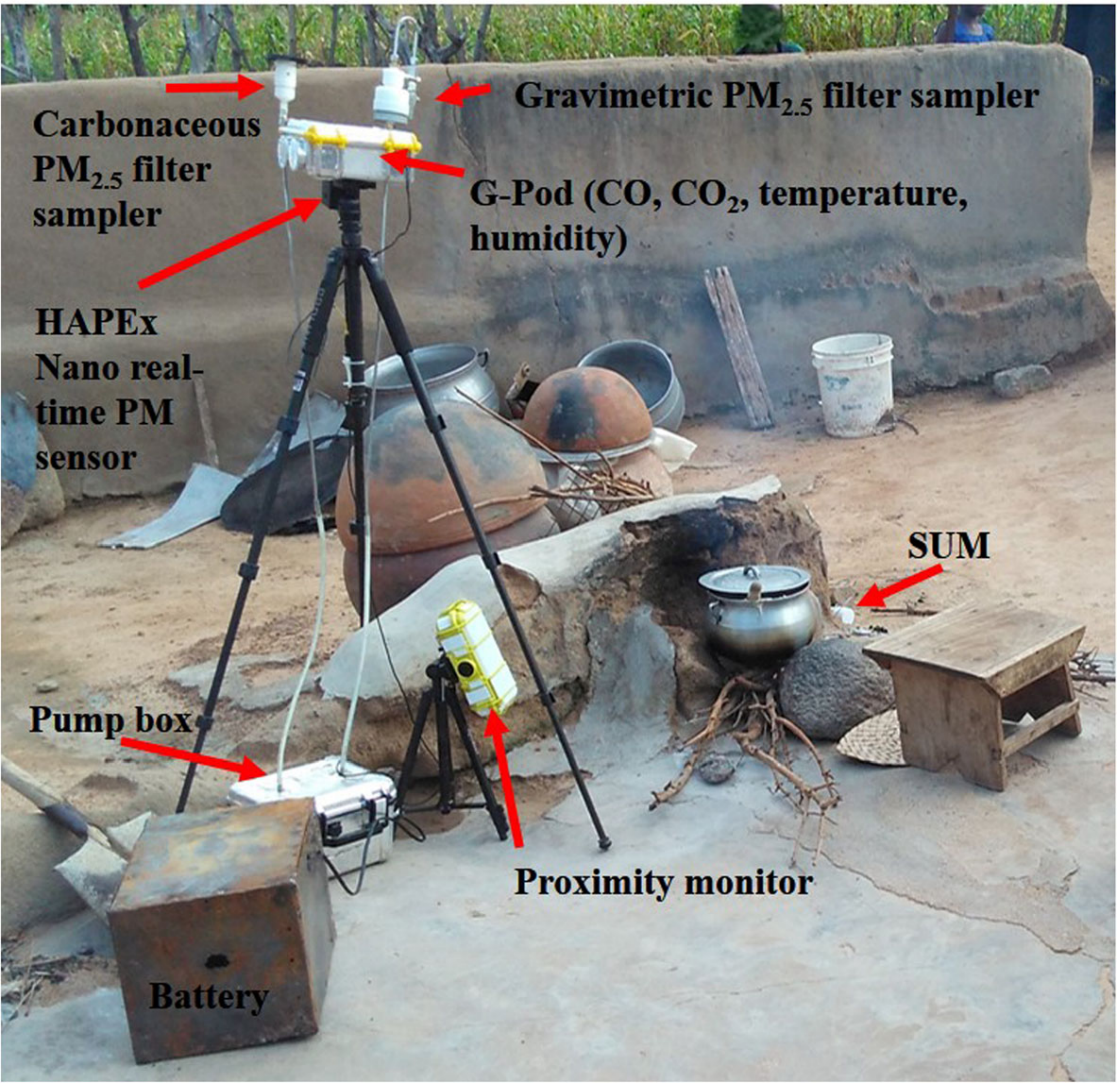
Monitoring equipment arrangement at an outdoor cooking area with a three stone fire. Images source: study authors

During each deployment period, one of the four households (rotating across the groups) is selected to participate in a series of supplementary measurements conducted in the primary kitchen. This focus household receives a G-Pod (mobilesensingtechnology.com) which is positioned one meter away from and one meter above the most used cookstove [33]. The G-Pod is a custom air quality sensor platform which measures CO, CO_2_, temperature, humidity and pressure at sub-minute intervals and is integrated with two HAPEx Nanos for measurements of kitchen level PM_2.5_ (Fig. 6). Lastly, the G-Pod is outfitted with two pumps and filter sampling trains to measure PM_2.5_ mass and carbonaceous PM_2.5_. Train one is composed of a cyclone inlet (URG-2000-30EQ) followed by a filter holder (URG-2000-30FG) containing a 47 mm PTFE filter (Teflo FPTPMP247) and then a pump (SKC PCXR8) adapted to pull a constant volume (3.0 ± 0.02 Lpm) of air for 48 h. Train two incorporates an impactor (URG-2000-30PASS-1), then a filter holder (URG-2000-25F-2-2.5) containing a 25 mm quartz fiber filter (Pall Tissuquartz 7200), and subsequently a pump (SKC AirLite) adapted to pull a constant volume (2.0 ± 0.02 Lpm) of air for 48 h. The G-Pod and filter sampling trains are powered by a 12-V car battery and last a full week without recharging. The pumps are both housed in a protective case with reflective surfaces to reduce overheating (see Fig. 6). The duration of the pump sampling is recorded by one pump (SKC PCXR8) and a custom PCB located within the pump enclosure acts as a redundant measure of total elapsed time.

#### Emissions measurements

Building on the REACCTING work, the research team has been collecting a set of emissions samples from multiple different types of sources in the study area. These samples help to characterize emissions from other sources, beyond residential cooking, that contribute to ambient air pollution and personal exposures. These include samples from the following sources: commercial cooking, traffic, kerosene lighting, trash burning, charcoal making, bush burning, diesel generators, and pito (local fermented beverage) brewing. These will continue with the goal of informing the pollutant exposure apportionment and cooking behavior study activities.

### Analysis and integration

To analyze stove purchase outcomes, we will estimate models of stove demand using discrete choice econometric methods [53], based on the experimental design described above. This analysis will be used first to statistically test the basic hypothesis that higher stove prices lead to lower demand for the stoves, all else equal (i.e. the ‘Law of Demand’ in economics). We also hypothesize that the higher-quality stoves will be in higher demand and that more of one or both stoves are weakly preferred, ceteris paribus.

After testing these basic hypotheses, we will then examine between-cluster heterogeneity in stove demand to investigate a primary research question of this study: whether households in the S1 group, previously exposed to peers with improved cookstoves, have statistically different demand for stoves compared to the S2 group. Formally, this will be tested first by jointly estimating stove demand using conditional logit and other discrete choice models for both groups. By interacting an indicator for S1 and S2 group assignment with stove model and price coefficients in the regression analysis, we will examine whether being in the peer group affects demand by shifting it up or down, or by changing price elasticity (i.e. making the demand curve flatter or steeper). While assignment to S1 and S2 groups is random, by virtue of the previous REACCTING study, we also include household characteristics collected from surveys in these demand models, to improve statistical efficiency of the analysis. We will also estimate mixed logit and latent class discrete choice models, to test whether unobserved preference heterogeneity is a statistically significant factor driving stove demand, and whether this preference heterogeneity is altered by S1/S2 group assignment.

Stove demand models will be estimated using both *initial stove orders* and *completed stove purchases*, with the understanding that some households who order stoves initially may ultimately be unwilling or unable to complete their purchases. If defaults are common, we will analyze factors associated with this outcome, including stove price, peer contact, and socioeconomic status, among other variables. In addition, we will conduct similar analyses to assess whether prices, peers, and/or perceptions affect stove *use* (measured by surveys and SUMs).

**Fig. 7:**
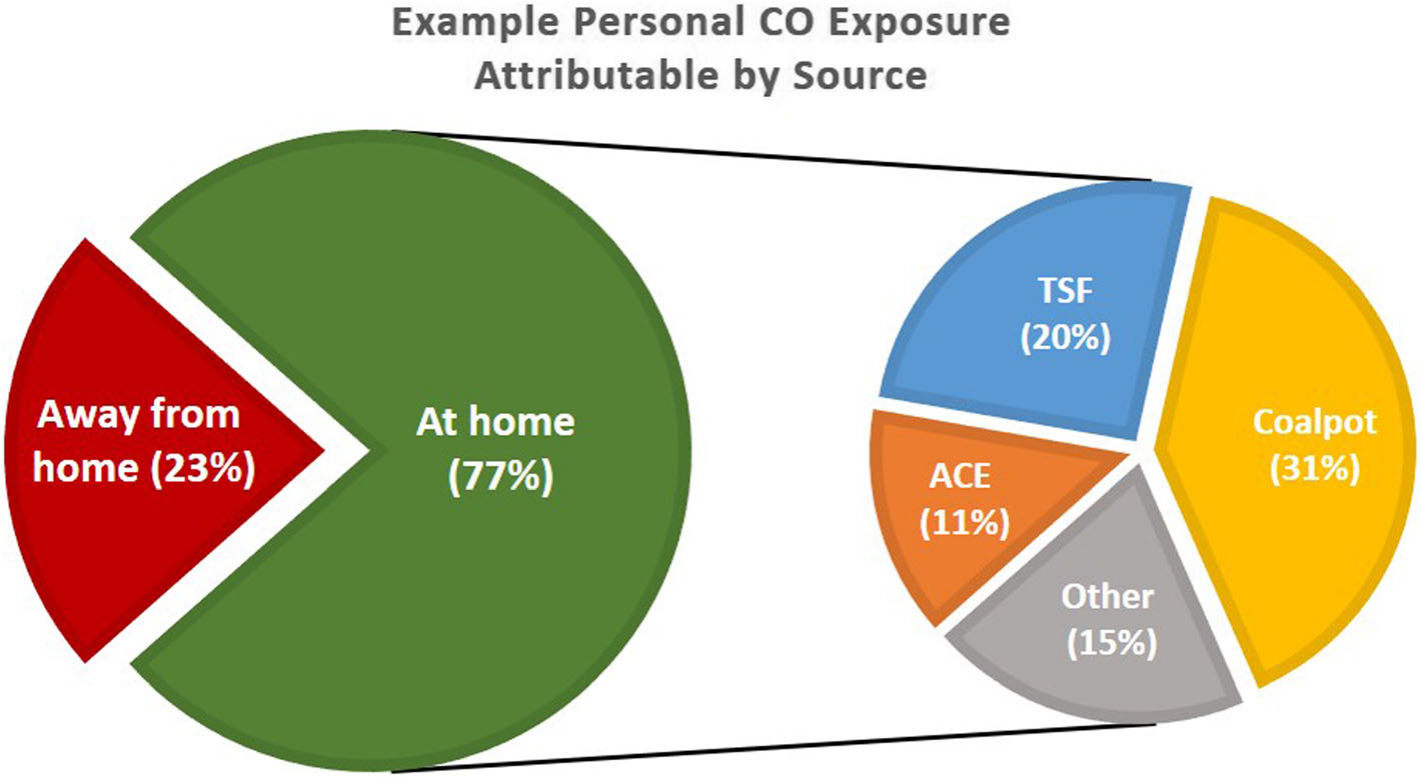
Example cumulative CO exposure (pie) with source contributions (slices) identified

From an air quality perspective, there are three main objectives to the analysis: (1) apportion CO and PM_2.5_ exposure to various cooking and non-cooking related activities at and away from the household (see Fig. 7), (2) estimate what effects the improved cookstoves have on CO and PM_2.5_ exposure across groups in addition to comparing within- and between-household variation, and (3) investigate relationships between mixing ratios of various combustion species (CO/CO_2_, CO/PM_2.5_) at the personal and microenvironmental levels to inform models estimating cumulative exposure [see 43]. These three objectives depend on contemporaneously measured stove usage, time-activity and exposure data at the various study households.

**Fig. 8:**
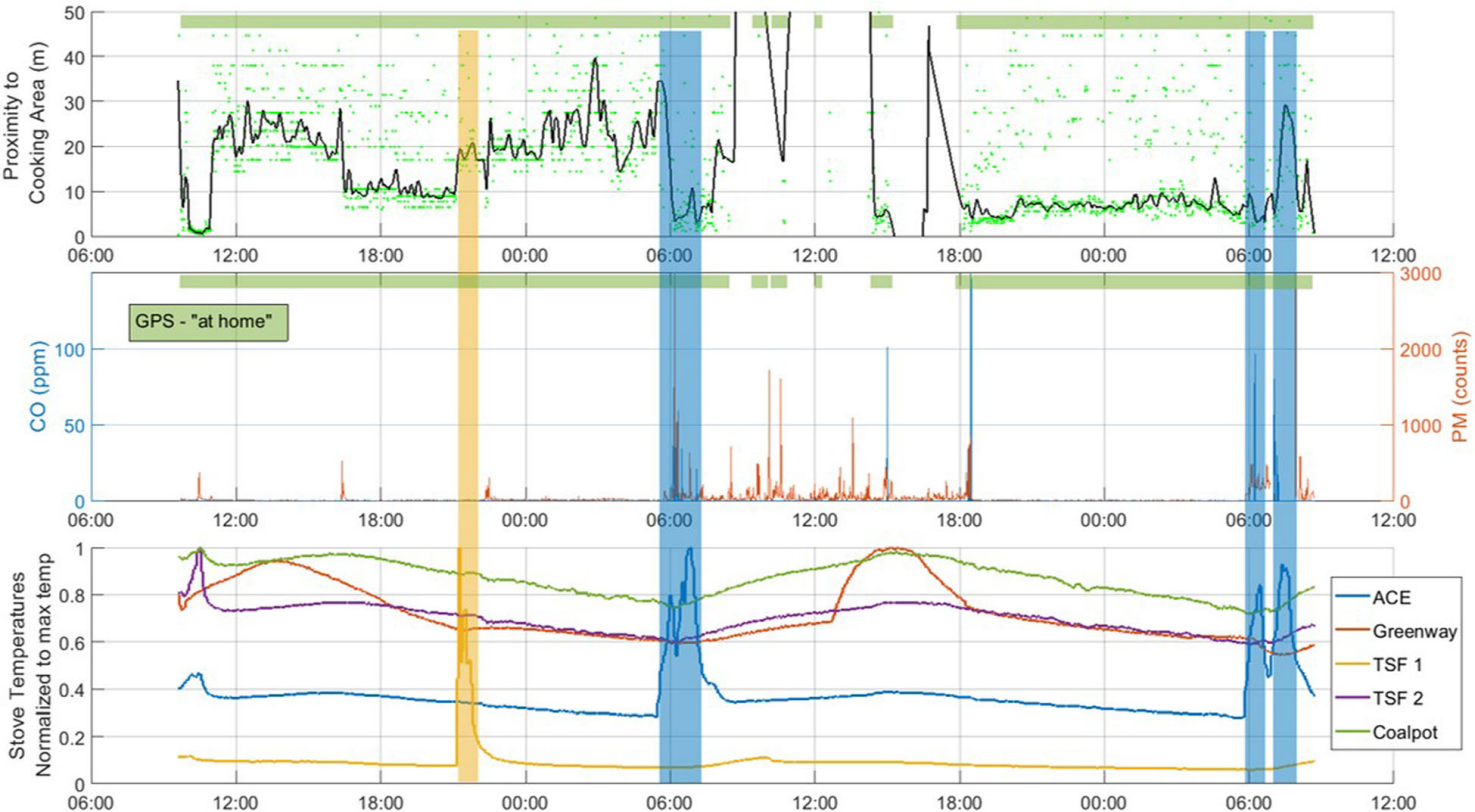
Preliminary data from a 48-h air quality household deployment. Pane a) shows user proximity to the nearest cooking area monitored with “at-home” status indicated by the green band. Pane b) shows CO and PM exposure with pane c) stove usage data for the five stoves located at this household (normalized to individual-stove maximum temperature)

To briefly illustrate the synthesis of these data, Fig. 8 depicts preliminary data from a 48-h deployment timeseries indicating measurements of a) participant proximity to the nearest cooking area and GPS location classification (at home or away, green band) with b) PM and CO exposure and c) stove usage for all five cookstoves at this household (normalized to individual-stove maximum temperature). The colored bands indicate a stove usage event and extend vertically to highlight the concurrently measured user proximity and exposure. Exposure incurred at moments in which participants are near a stove, and that stove is in use (see blue band on left) can be apportioned appropriately. Contrast this with the interval marked by the yellow band representing a clear three stone fire event, however, the proximity data indicates the participant is not near the stove and has minimal incurred exposure.

To advance the analysis, we plan to perform a comprehensive uncertainty assessment of each data stream including a rigorous in-field photo validation of stove use and proximity, a task seldom pursued in large scale interventions. We also aim to improve the explanation of variation between mean HAPEx Nano measurements and cumulative gravimetric PM_2.5_ measurements with the inclusion of temperature, humidity, pressure, kitchen level CO and CO_2_ concentrations as well as the sample chemical composition (e.g. elemental carbon, organic carbon) from speciated PM_2.5_.

A final type of integration involves using both survey and measurement data to examine relationships among stove perceptions, stove use, and stove performance. For example, we will assess whether households that perceive stoves to be cleaner actually use their stoves more and/or experience reduced exposure to pollutants.

Results will be disseminated through peer reviewed publications and scientific conferences, as well as through community meetings with study participants.

## Discussion

The P3 project seeks to provide a much needed integration of approaches to understanding the drivers and impacts of public health-related technology adoption. In many prior cookstove studies, epidemiologists have sought to measure the health effects of cleaner cooking devices, only to be confronted with the problem of “imperfect compliance”: that is, households’ failure to switch to exclusive use of the cleaner stoves [e.g., 54]. This confounder, from the perspective of epidemiologists, is the main research question for social scientists: how and why do people decide to adopt and use a new technology? To fully understand the potential real-world public health effects of stoves and similar technologies, an interdisciplinary approach that embeds rigorous social science methods is needed.

The P3 study is uniquely positioned to address a policy relevant set of research questions in the context of public health technology adoption. Despite widespread acknowledgement that prices, peers, and perceptions are key drivers of households’ decisions to adopt new technologies and behaviors, rigorous study designs testing the separate and interacting effects of these factors have been elusive. By taking advantage of the randomized introduction of free stoves in the REACCTING project, we aim to fill this gap.

Our project integrates state-of-the-art social science and exposure analysis methods. Our experimental methods are rooted in economic program evaluation approaches, and incorporate stated and revealed preference methods to inform our price levels within the experiment. Notably, our inclusion of survey measures capturing users’ perceptions of stoves over time will allow us to build better theoretical models of the adoption process by clarifying mechanisms linking prices and peer effects to adoption outcomes. In other words, we hope to shed light on why prices and peers shape technology choice, with implications for policy design.

Simultaneously, our exposure measurement approaches enable a more complete assessment of the linkages between stove use, emissions and exposure outcomes. The additional information offered from time-activity measurements (proximity and GPS) in concert with stove usage data enables us to apportion exposure to various at-home sources (e.g. stoves, kerosene lamps, trash burning etc.) while allowing us to identify exposure incurred outside of the home. Breaking the cumulative exposure “pie” into “slices” of source-identified contributions will enable us to disentangle cooking-related exposures, facilitating direct comparisons across stove groups and individual stove (and fuel) types. Likewise, we are well poised to explore how cooking behavior (e.g. cooking duration, proximity/tending to stove) may be affected by new stove technology adoption.

The integration of these approaches in the P3 project allow us to examine how and why technology adoption occurs and how these processes influence exposures, with key implications for public health policy and practice.

## Additional file

Additional file 1: Informed Consent statement for the P3 project. (PDF 667 kb)

## Abbreviations

CO: Carbon Monoxide
FGD: Focus Group Discussion
HAP: Household Air Pollution
HDSS: Health and Demographic Surveillance Survey
KND: Kassena-Nankana Districts
NHRC: Navrongo Health Research Centre
ODK: Open Data Kit
ORGIIS: Organisation for Indigenous Initiatives and Sustainability Ghana
P3: Prices, Peers, and Perceptions
PM_2.5_: Particulate Matter less than 2.5 μm in diameter
REACCTING: Research on Emissions, Air quality, Climate, and Cooking Technologies in Northern Ghana
RESPIRE: Randomized Exposure Study of Pollution Indoors and Respiratory Effects
SUM: Stove Use Monitor
WTP: Willingness to Pay
