# Supplementary material for: Prices, peers, and perceptions (P3): study protocol for improved biomass cookstove project in northern Ghana
Source: BMC Public Health. 2018 Oct 29;18:1209. doi: 10.1186/s12889-018-6116-z (PMC6206711; doi:10.1186/s12889-018-6116-z)
Supplement: Supplementary file 1 — Informed Consent statement for the P3 project. (PDF 667 kb) [file 12889_2018_6116_MOESM1_ESM.pdf]

Prices, Peers, and Perceptions (P3): Study Protocol for Improved Biomass Cookstove Project in Northern Ghana

Appendix: INFORMED CONSENT - HOUSEHOLDS

INTRODUCTION

Hello, my name is ______. I am working with the Navrongo Health Research Center, and the University of Colorado and North Carolina State University in the United States. We are doing a study about cooking stoves and other issues in this area. We do not plan to talk to all residents in this area, but have selected several to participate in this study. You are one of those selected to participate, if you are willing. **I would like to speak with the person in this household who does the most cooking.**

STUDY PROCEDURE

We are interested in learning about how people cook in this area. If you agree to participate in this study, we will visit you about four times over the next two years to do household surveys. These surveys will take approximately one hour to complete.

In some households, we will want to measure how much smoke from cooking is in the air you and other household members breathe and how this changes from day to day. To measure this smoke exposure, we will ask you to wear an electronic monitoring device and a filter sampler for 24 to 48 hours every three months for the next two years. The ELECTRONIC DEVICE is the size of a mobile phone and measures and records the amount of smoke-related pollution it is exposed to. This device is worn on the shirt or clipped to pants. The FILTER SAMPLER collects small particles from the air on a filter. This device includes batteries and a pump that need to be stored in a small bag and makes a quiet humming sound. These methods have all been found to be extremely safe approaches to measuring air pollution. The electronic instruments could possibly break, causing smoke or fire, but the risk of this happening is very small.

In a smaller number of households, chosen from the group that participates in the smoke exposure monitoring, we would like to monitor what times stoves at your household are lit and when the participant wearing the ELECTRONIC DEVICE is nearby these stoves. We will accomplish this by setting up two CAMERAS equipped with special lights for night vision approximately 10 feet away from each of your household's two main cooking areas. These cameras will take a photograph of each cooking area, and any person in the cooking area, approximately every 30 seconds for the entire 48 hour deployment of the smoke exposure equipment described above. The night vision should not disturb you, as this feature uses dim red lights. After we collect these photographs, a researcher will go through all pictures and determine at which times (1) any stove in the cooking area was lit and (2) the participant wearing the ELECTRONIC DEVICE was in the cooking area. After recording these times, all pictures will be deleted immediately and used for no other purpose.

Finally, as part of this study you may have the opportunity to buy new stoves. More information about these stoves and their prices will be provided in a meeting that will be held at LOCATION on DATE at TIME. If you agree to participate in the study, you or another household member will be asked to attend this meeting. However, buying a stove or stoves is completely voluntary, and you may participate in this study even if you choose not to buy a stove.

VOLUNTARINESS

Taking part of this study is completely voluntary. You have every right to refuse to participate. If you should refuse, you will not suffer any consequences.

Additionally, you have the right to refuse to participate in the CAMERA portion of this study while still participating in the remainder of the study. In this case, you will not suffer any consequences and we will continue with the remaining activities in this study.

WITHDRAWAL

If you chose to participate in this study, you have the right to withdraw from it at any point in time without any consequences to you (or your child/ward). You can also refuse any study procedure that you are not comfortable with. *You are free to skip any questions you do not wish to answer or to stop at any time. You may ask the researchers any questions you have at any time.*

COMPENSATION

You will not be paid for your participation in this study.

CONFIDENTIALITY

We will need to connect your name to the information you give us, but only for a short time—while we are gathering information from many households. After that, we will save the information and report what we learn using numbers, not names. Only we, the researchers, will ever see the surveys with people's names.

QUESTIONS

If you have any questions concerning the study, you can contact Dr. Abraham Oduro at the Navrongo Health Research Centre or call him at 0244593231.

The Navrongo Health Research Centre Institutional Review Board and the Ghana Health Services Ethics Review Committee has reviewed and given approval for this study to be conducted. If you have questions with regards to your rights as a participant in the study, you can contact the Chairmain, Dr. Koku Awoonor-Williams on telephone number 0244564120.

ORAL CONSENT OF PARTICIPANT

(Asked to ALL households)

Do you agree to participate in this study?

- ☐ Yes
- ☐ No

(Asked to households selected for CAMERA monitoring)

Do you also agree to participate in the CAMERA monitoring portion of this study?

- ☐ Yes
- ☐ No
